# Executive and Motor Functions in Older Individuals with Cognitive Impairment

**DOI:** 10.3390/bs12070214

**Published:** 2022-06-28

**Authors:** Maria Chiara Fastame, Ilaria Mulas, Valeria Putzu, Gesuina Asoni, Daniela Viale, Irene Mameli, Massimiliano Pau

**Affiliations:** 1 Department of Pedagogy, Psychology, Philosophy, University of Cagliari, Via Is Mirrionis 1, 09123 Cagliari, Italy; 2 Department of Mechanical, Chemical and Materials Engineering, University of Cagliari, 09123 Cagliari, Italy; imulas89@gmail.com (I.M.); massimiliano.pau@unica.it (M.P.); 3 Center for Cognitive Disorders and Dementia, Geriatric Unit (ASL Cagliari District), SS. Trinità Hospital, 09121 Cagliari, Italy; valeria.putzu@atssardegna.it (V.P.); gesuina.asoni@atssardegna.it (G.A.); danielaviale@tiscali.it (D.V.); dott.irenemameli@gmail.com (I.M.)

**Keywords:** aging, executive functions, handgrip, cognitive impairment, functional mobility, motor skills, older adults, late adulthood, muscular strength, gait

## Abstract

Background: A current research trend is the examination of the interplay between cognitive functioning, higher-order processes, and motor efficiency in late adulthood. However, the association between motor and cognitive functions when cognitive decline occurs has not been extensively explored. This study investigated whether gait features, functional mobility, and handgrip strength were associated with executive functions in older people with mild cognitive impairment (MCI) or dementia. Methods: 127 older participants (M_age_ = 77.9 years, SD = 5.8 years) who had received a diagnosis of MCI and dementia voluntarily took part in the study. A battery of tests assessing global cognitive function, executive functions, muscular strength, functional mobility, and spatio-temporal parameters of gait was completed by the participants. Results: Statistically significant correlations were obtained between global cognitive function, executive functions, and motor efficiency measures. Moreover, a series of regression analyses showed that 8–13% of the variance of several motor parameters was predicted by several executive functions. Additionally, walking, functional mobility, and global cognitive function predicted 53–71% of the variance relative to the occurrence of dementia. In conclusion, motor functioning is closely related to cognitive functioning in late adulthood. Conclusions: The assessment of muscular strength and functional mobility should be promoted in clinical settings.

## 1. Introduction

The last decades of life are often accompanied by progressive decline in cognitive processes and deterioration in functional health (e.g., postural stability, locomotion), due to normal biological factors or age-related pathological conditions [1,2]. There is evidence that in advancing age functional health is closely related to cognitive efficiency [3], such that declining gait speed has been considered a precursor of cognitive deterioration in late adulthood [4,5]. It has been argued that the occurrence of cognitive impairment in the geriatric population causes gait to be slower, increases gait variability and fall risk and reduces gait stability [6]. Both complex processes, such as postural control (i.e., the ability to control the position of the body—necessary to prevent the risk of fall), and new motor program learning, involve higher-order cognitive processes comprising executive functions (EF). Despite the lack of a commonly accepted definition, in this context, EF refers to a set of “general purpose control mechanisms that modulate the operation of various cognitive subprocesses and thereby regulate the dynamics of human cognition” ([7], p. 50). These central processes encompass shifting (i.e., the capacity to switch flexibly from one cognitive task to another or from one mental set to another), response inhibition (i.e., the capacity to suppress irrelevant outputs), updating (i.e., the capacity, firstly, to monitor and code mental representations in working memory and then to revise these internal representations, replacing the old information with newer and more appropriate information), self-regulation (i.e., the ability to control one’s behavior through self-monitoring, self-evaluation, and self-reinforcement), sequencing, strategic planning, sustained attention (i.e., the ability to maintain the focus of attention on a certain task for a prolonged time interval), divided attention (i.e., the ability to direct one’s attention to perform two or more tasks concurrently), processing speed and goal-directed behavior [7,8], for a review see [9]. EF are handled by a high-order control structure, i.e., the central executive (Baddeley and Hitch [10]), or the supervisory attentional system (Norman and Shallice [11]), that is driven by complex distributed neural networks engaging different brain structures, including the frontal and parietal cortical areas, the thalamus and subcortical (i.e., basal ganglia) regions [12].

Research conducted with typically developing older individuals has demonstrated a significant reduction in EF (e.g., processing speed, set-shifting, goal-directed behavior, divided and selective attention) efficiency which is mediated by a gradual decrease in brain volume (e.g., in the right prefrontal and parietal cortices) [13]. Additionally, in normal aging, executive functioning (e.g., response inhibition, motor control) is associated with functional and life-space mobility [14,15] and greatly benefits from aerobic fitness training, as this contributes significantly to maintenance of prefrontal cortex volume and prevents tissue loss in cerebral regions engaged by EF [16,17]. In contrast, in atypically developing cognitive conditions (i.e., mild cognitive impairment, MCI and Alzheimer’s disease, or other forms of dementia), advancing aging is characterized by the co-occurrence of executive functioning deterioration and loss of motor proficiency, which reflects significant atrophy of brain regions engaged by EF. In this regard, it has been documented [18] that, compared to cognitively intact older individuals, patients who were diagnosed with MCI and pre-MCI (i.e., the intermediate state between healthy aging and MCI, with self-reported symptoms of cognitive impairment which are not confirmed by objective neuropsychological assessment) exhibited lower left-sided hippocampal volumes associated with EF deficits (i.e., semantic fluency, set-shifting, inhibition, reduced speed of processing). Further recent evidence suggests that demented older individuals display more aberrant motor behaviors and inhibition problems than patients with MCI because the former group undergoes extended atrophy of frontal areas of the brain [19]. However, according to Jung et al. [20], older individuals with MCI combined with memory and EF dysfunctions, reflecting significant cortical atrophy (i.e., especially a thinner frontal cortex) are at higher risk of developing dementia and more diffuse cortical thinning one year later. Consistent with these observations, according to Duara et al. [18], set-shifting scores of MCI and pre-MCI patients recorded at baseline contribute significantly to predicting the occurrence of dementia three years later.

Further research has highlighted that older individuals with cognitive deterioration are also impaired in motor behavior, particularly with respect to gait and functional mobility. Although gait alterations are commonly observed in older adults as a result of the physiologic aging process, specifically in relation to reduction in speed, stride length, and cadence, as documented by Prince, Corriveau, Hébert, and Winter, [21], recent studies have reported that further anomalies may occur in the presence of cognitive impairments. Moreover, early disturbances in cognitive processes, such as working memory (i.e., including EF), often coexist with slower gait speed and instability [22,23].

Similarly, functional mobility (i.e., a term which encompasses several motor tasks necessary to perform common activities of daily living, such as rising from and sitting down in a chair, changing direction, etc.) has been found to be linked with cognitive performance. Several studies have reported that the performance in the timed-up-and-go (TUG) [24] test, which is widely employed to assess functional mobility and risk of falls in community-dwelling and frail older adults [25,26], is worse among individuals with cognitive impairments [27,28]. Moreover, moderate to large correlations have been observed between the overall time necessary to complete the TUG test and cognitive performance, assessed using either Addenbrooke’s Cognitive Examination Revised ACE-R [29,30] or the Montreal Cognitive Assessment (MoCA) [31]. Additionally, it is noteworthy that it has been suggested that TUG time might represent a valid marker to support the diagnosis of dementia, including the prodromal phase [32].

Another trend in burgeoning research has been the investigation of the interplay among cognitive decline, EF, and motor efficiency in late adulthood, since the brain regions engaged in EF tasks are crucial to performing motor responses (e.g., the dorsolateral prefrontal cortex encodes motor planning; the basal ganglia and cerebellum contribute to the execution of movement sequences). In this regard, it is well-established that EF dysfunction is related to an increase in fall rates both in cognitively intact [3,33] and impaired older individuals [34]. Thus, a body of research has documented that a general deficit of EF [29,30], as well as specific decreases in inhibition, set-shifting, verbal fluency, and processing speed, predict deficit in functional mobility (i.e., assessed via the TUG task) and of people with MCI and early-stage dementia [30,35,36]. Additionally, Persad et al. [37] found that cognitive flexibility, problem-solving, and planning of cognitively deteriorated individuals correlated with walking speed. According to the authors, patients with MCI and executive dysfunction, and those with dementia, must perform complex motor tasks requiring decision-making and cognitive flexibility, and the risk of choosing the incorrect motor response is higher than for cognitively healthy older people. Moreover, it has also been found that poorer speed of processing was related to a steeper deterioration in gait speed [2]. Extending this, Camargo et al. [38] documented that decline in the speed of processing, number sequencing abilities, and set-shifting tasks predicted a significant decline in gait speed and muscular strength (assessed by means of isometric handgrip strength, HGS) in older individuals developing dementia.

## 2. Background

Although the efficiency of motor and cognitive functions is crucial for the maintenance of quality of life, including in moderately deteriorated older people, to date, to our knowledge, no studies have thoroughly investigated the association between EF and upper and lower extremity motor skills in older individuals exhibiting different degrees of cognitive deterioration. Thus, this study sought to determine whether, in a sample of cognitively impaired participants, motor proficiency and executive functioning are associated. To this end, we identified three complementary objectives: (1) to investigate whether gait speed, functional mobility, and HGS measures are associated with global cognitive efficiency and distinct EF in cognitively impaired aged individuals; (2) to explore if EF predicts HGS and mobility measures in a sample of cognitively deteriorated individuals; (3) to examine whether strength and mobility parameters account for the type of cognitive deterioration (i.e., MCI vs. dementia), when the impact of body mass index (BMI), gender, and global cognitive efficiency are controlled for.

Based on previous literature, global cognitive function was expected to be associated with gait speed [2,4,5], TUG [30], and HGS [38] measures. Significant positive associations were also expected between speed of processing and TUG performance [35] and gait speed [2]. In addition, verbal fluency was expected to be related to TUG performance [30], whereas action planning was hypothesized to be related to gait speed [37]. Speed of processing and set-shifting were expected to predict TUG performance [35,36], HGS, and walking speed [38]. Finally, MCI and dementia were expected to be predicted by HGS [39,40], walking speed [41,42], and TUG time [32].

## 3. Materials and Methods

### 3.1. Participants

One hundred and twenty-seven elderly individuals (M_age_ = 77.9 years, SD = 5.8 years), comprising 79 females and 48 males, were enrolled at the Center for Cognitive Disorders and Dementia, a public service provided by the Italian National Health System located in the city of Cagliari (Italy), where they received a diagnosis of cognitive impairment (i.e., MCI vs. dementia).

Participants were eligible if they were: (1) community-dwelling and resident in the metropolitan area of Cagliari; (2) diagnosed as cognitively impaired (i.e., MCI or dementia disorders); (3) free from neurologic (i.e., Parkinson’s disease, multiple sclerosis and stroke) and orthopedic conditions interfering in mobility; and (4) capable of walking independently without the need of any support, such as walking frames, canes, crutches.

Consistent with previous research (e.g., [43]), formal schooling was dichotomized as low (i.e., ≤8 years, *n* = 90) or high (i.e., ≥9 years, *n* = 37). Gender (χ^2^ = 7.57, df = 1, *p* = 0.006) and education (χ^2^ = 22.1, df = 1, *p* < 0.001) were not counterbalanced across the participants. However, cognitive impairment (i.e., MCI vs. dementia) was counterbalanced across the male and female groups (χ^2^ = 0.975, df = 1, *p* = 0.323).

### 3.2. Materials

Each participant completed the following battery of psychological tasks:

The Mini-Mental State Examination (MMSE) [44] was used as a screening test to assess general cognitive efficiency. It includes 18 items assessing different cognitive domains, such as mental calculation, attention, orientation, and immediate and delayed recall. Scores were adjusted for educational attainment and age (maximum total score = 30), consistently with the norms proposed by Magni et al. [45].

The clock-drawing test [46] (Italian validation [47]) is a pencil and paper visuo-spatial tool that was used to assess planning abilities, motor sequencing, task monitoring, and goal-directed behavior skills. Each participant was asked to draw the hands of a clock at 2:45 on a pre-drawn circle. Based on the scoring system proposed by Mondini et al. [47], each drawing clock was rated assigning a score ranging between 0 and 10, where 0 indicated the worst performance and 10 the best.

A semantic fluency test [48] was used to assess the efficiency of three distinct executive functions: self-monitoring, set-shifting, and inhibition. Each participant was invited to recall as many words as possible belonging to certain semantic categories (e.g., animals) in a certain time interval, avoiding generating the same word more than once. Thus, to perform this task, each participant had to access his/her mental lexicon, and he/she had to select the appropriate stimuli and inhibit the irrelevant stimuli. Three distinct categories were proposed. The total number of correct responses was computed. The final score was corrected for age and years of education.

The verbal fluency subtest of the ACE-R battery (ACE-R-Fluency) [49](Italian validation [50]) was used to assess the efficiency of self-monitoring, set-shifting, and inhibition. Each respondent had to name as many words as possible belonging to a category (i.e., semantic fluency) or starting with a certain letter (i.e., phonological fluency). As suggested by the authors, a combined total correct score was calculated.

The Trail Making Test-Part A (TMT-A) [51] (Italian validation [52]) was used to assess the efficiency of motor speed, visual scanning, and number sequencing abilities. Each respondent had to connect 25 encircled numbers distributed on an A4 sheet in ascending numerical order, without lifting the pen from the paper as accurately and as quickly as possible. The total time (expressed in seconds) taken to perform this task was computed.

The Attentional Matrices Test [53] is a verbal task that was administered to assess the efficiency of selective and divided attention and that also requires set-shifting. For each of the three matrices, the participant had to select a series of stimuli targets (i.e., digit numbers) as soon as possible within a time limit of 45 s. The total number of correct responses was recorded.

The gait and TUG tests were carried out by means of a wearable inertial sensor (G-Sensor^®^, BTS Bioengineering S.p.A., Milan, Italy), previously employed for similar investigations in older adults [54,55,56,57]. The sensor was attached to the individual’s trunk, using a semi-elastic belt, at two different positions, which approximately corresponded to S1 vertebrae (for gait analysis) and L1 vertebrae (for the TUG test) locations. Spatial-temporal parameters of gait, including speed, step length, and cadence, were obtained by processing trunk accelerations along antero-posterior, medio-lateral and vertical directions collected by the sensor while the participant walked at a self-selected speed on a 30-m straight trajectory. The TUG test was carried out by asking participants, sitting on a standard office chair, to stand up, walk for 3 m at a comfortable and safe speed [24], perform a 180° turn around a cone, walk back to the chair and perform a second 180° turn to sit down and end the test. The trunk accelerations acquired by the sensor were processed to calculate the overall TUG time and the times associated with each sub-phase (i.e., sit-to-stand, intermediate and final 180° turning, and stand-to-sit).

The HGS measurement was carried out by means of a validated [58] digital hand dynamometer (DynEx, MD Systems, Westerville, OH, USA), previously employed in studies involving older adults [55,59]. The HGS score representative of a certain participant was defined as the maximum value obtained from six trials (three for each limb) alternated and interspersed by 20 s of rest.

### 3.3. Procedure

This investigation was conducted in accordance with the ethical standards of the institutional research committee and with the 1964 Helsinki Declaration and its later amendments. Written informed consent was obtained from all participants before being individually tested in a quiet room of the Center for Cognitive Disorders and Dementia.

First, the psychosocial measures were presented, and immediately after the motor tasks were proposed in the same experimental session. After that the MMSE was presented, the presentation order of the further psychological measures was counterbalanced across the participants. Accordingly, the presentation order of the motor tasks was counterbalanced across the participants. Overall, each experimental session lasted approximately 50 min.

## 4. Results

First, Pearson’s product-moment coefficients were calculated to check for multicollinearity and to explore the degree of association between global cognitive efficiency (i.e., MMSE score), the clock-drawing, verbal fluency, TMT-A, Attentional Matrices measures, and HGS, walking speed, step length, and TUG sub-phase times. The outcomes are presented in Table 1.

As illustrated in Table 1, the associations among the variables was not considered high (i.e., r ≥ 0.9); therefore, multicollinearity among the variables was excluded. Based on the results of the correlational analyses, a series of stepwise regression analyses was conducted to explore whether distinct EF aspects (i.e., semantic fluency, ACE-R-fluency, clock and TMT-A measures) predicted HGS, walking and TUG measures. Preliminary analyses were conducted to ensure that there was no violation of the assumptions of normality, linearity, and homoscedasticity. Moreover, tests to assess if the data met the assumption of collinearity indicated that multicollinearity was not a concern. It was found that semantic fluency predicted approximately 4% of the variance in TUG-Intermediate-turn (adjusted R^2^ = 0.038, F (1, 82) = 4.21, *p* = 0.04, B = 0.18, SE = 0.09, β = 0.221, 95% CI [0.05, 0.35], t = 2.05, *p* = 0.04) and in step length (adjusted R^2^ = 0.037, F (1, 83) = 4.12, *p* = 0.04, B = −0.23, SE = 0.112, β = −0.22, 95% CI [−0.452, −0.005], t = −2.03, *p* = 0.04) assessments, respectively. Moreover, approximately 4% of the variance in the TUG-Final-turn assessment was predicted by the TMT-A index (adjusted R^2^ = 0.044, F (1, 81) = 4.75, *p* = 0.03, B = 0.225, SE = 0.103, β = 0.235, 95% CI [0.02, 0.431], t = 2.18, *p* = 0.03). Additionally, 9% of the variance relative to the HGS (adjusted R^2^ = 0.089, F (4, 79) = 3.05, *p* = 0.02) was predicted by the TMT-A score (B = −0.372, SE = 0.125, β = −0.35, 95% CI [−0.622, −0.123], t = −2.976, *p* = 0.004), whereas MMSE (B = −0.332, SE = 0.241, β = −0.181, 95% CI [0.812, 0.148], t = −1.378, *p* = 0.17), ACE-R-Fluency (B = −0.088, SE = 0.152, β = −0.181, 95% CI [−0.39, 0.213], t = −0.586, *p* = 0.56) and clock (B = 0.10, SE = 0.17, β = 0.088, 95% CI [−0.238, 0.437], t = 0.589, *p* = 0.56) scores were not significant predictors.

Finally, a logistic regression analysis was conducted to assess the impact of HGS, walking speed and step length, TUG-Sit-to-stand, and TUG-Stand-to-sit on the likelihood that the participants would report cognitive impairment (0 = MCI and 1 = dementia), while controlling for BMI, gender, and global cognitive efficiency. These covariate measures were added in the following order: first, the BMI index was inserted, gender was added in Model 2, and finally, the MMSE score was also included in Model 3. The full model containing all predictors was statistically significant, χ^2^ (8, *N* = 127) = 93.8, *p* < 0.001, indicating that the model was able to distinguish between respondents with MCI and those with dementia. The model as a whole explained between 52.5% (Cox and Snell R^2^) and 70.7% (Nagelkerke R^2^) of the variance in cognitive impairment status, and correctly classified 84.1% of cases. However, when the impact of the covariates was controlled for, several lower extremity motor measures (i.e., walking speed m/s, TUG-Sit-to-stand, and TUG-Stand-to-sit) contributed to explaining between 6% (Cox and Snell R^2^) and 8% (Nagelkerke R^2^) of the variance. Table 2 illustrates the statistical contribution of the factors predicting the occurrence of cognitive impairment.

## 5. Discussion

According to the World Health Organization, approximately 115.4 million people will exhibit dementia by 2050; therefore, cognitive deterioration represents an important challenge for the maintenance of quality of life in aged individuals. A further challenge is the identification of the psychological and functional (i.e., motor) markers of cognitive deterioration, to enable screening and preventive intervention programs to promote the independence of people at risk of severe dementia. In this regard, the study of the relationship between motor and EF efficiency in cognitively impaired older people is crucial, since functional deficits disrupt physical independence and predict morbidity, and even mortality, in later adulthood [60].

This study was aimed mainly at clarifying the association between motor proficiency and EF in cognitively impaired aged individuals. Overall, focusing on the results of the correlational analyses, the current findings extend previous evidence documenting that cognitive and functional health are associated and play a crucial role in the maintenance of independence and the quality of life of aged individuals [1,4]. Three main conclusions can be drawn. First, it was found that global cognitive efficiency (i.e., MMSE score) and distinct EF (i.e., clock-drawing, semantic fluency, ACE-R Fluency, Attentional Matrix, and TMT-A measures) were significantly associated with strength (i.e., HGS) and mobility (i.e., TUG) proficiency parameters. As expected, general cognitive efficiency was associated with TUG performance [30] and HGS [38]. However, against our hypothesis, no significant relationships were found between MMSE score and gait speed, although the association between global general efficiency and step length approached significance. Moreover, as expected, speed of processing (i.e., TMT-A) was significantly associated with HGS and TUG performance [35], whereas no significant associations were found between the former and gait speed [2]. Additionally, consistent with de Melo et al. [30], verbal fluency was found to be related to TUG performance, whereas, against our hypothesis, action planning (i.e., the clock-drawing test) was not associated with walking (i.e., speed and step length) measures [37].

Furthermore, it was found that approximately 6–8% of the variance related to the occurrence of cognitive impairment was explained by certain lower extremity motor measures (i.e., walking speed, TUG Sit-to-stand, and TUG Stand-to-sit times). The strongest predictor of reporting a cognitive impairment problem was the TUG-Stand-to-sit time, with an observed odds ratio of 3.11. This indicated that participants who had dementia were over three times more likely to report a problem with the postural transition from standing to sitting than those who exhibited MCI, controlling for all other factors in the model. The few existing studies that have been carried out with accelerometers have reported that, in older adults, either physically unfit (i.e., frail) or affected by neurologic conditions, the stand-to-sit phase is characterized by significant differences in terms of duration and trunk accelerations/displacements in comparison to healthy individuals [61,62,63]. In our case, it is hypothesized that individuals with MCI exhibit what has been termed “cautious sitting” [63], that is, a stand-to-sit transition characterized by abnormally longer postural adjustment and preparation for sitting. Such a strategy is probably due to a combination of factors which include impaired postural control, reduced strength of the knee flexors, and fear of falling [63]. Therefore, as pointed out in previous studies, motor deficits in late adulthood are associated with executive dysfunction, which, in turn, disrupts everyday functioning, even in the performance of very routine adaptive behaviors (e.g., the activities of daily living, such as bathing) [64,65].

Finally, consistent with previous studies [30,36,38], motor speed, visual scanning, number sequencing abilities (i.e., assessed through the TMT-A), and self-monitoring, set-shifting, and inhibition (i.e., assessed through the verbal fluency tasks) skills predicted 4–9% of the variance relative to specific TUG, handgrip strength, and walking parameters.

Altogether, from an applied viewpoint, the findings of the present study suggest that the combined assessment of EF, gait, functional mobility and muscular strength in older individuals at risk of cognitive impairment or diagnosed with MCI or dementia, may provide clinically relevant information with important practical implications. It appears crucial to design specific training programs which can enhance both physical and cognitive determinants of functional mobility, to increase the possibility to prevent, as far as possible, the consequences of a significant reduction in independence in daily life. The mobility/strength measures used in the current investigation are ecologically valid, sensitive and easy to administer. Therefore, their use in the clinical assessment of the geriatric population should be encouraged, in combination with the usual psychological tests. The results of the regression analyses indicate that when different degrees of cognitive decline occur, older individuals may appear slow, clumsy, and may need some supervision or support to deal with their daily routine activities (e.g., initiating a task, maintaining attention on an activity for a certain time, shifting from one task to another) engaging EF, and motor responses, such as cooking, bathing, or cleaning their own home [64].

The study does have some limitations. The relatively small sample size and the short EF battery limit the generalizability of the findings. Furthermore, only community-based participants took part in the study; therefore, caution is needed to generalize these findings to older people living in nursing homes. Our participants were community-dwellers, physically active in their community, whereas, as reported in previous research, older people living in nursing homes tend to be sedentary, and to sit for long periods in their private rooms even if they can walk independently [66,67]. Therefore, the current investigation should be replicated with older individuals living in nursing homes. Finally, the developmental trends of the measured cognitive, motor, and EF functions have not been explored longitudinally. Therefore, future longitudinal studies, including the collection of multiple records of motor and EF proficiency, should be encouraged to examine developmental and maturational trajectories within the same individuals over time. Finally, it would be helpful if, in future, this study were replicated, including the administration of standardized measures of activities of daily living (ADL) and of instrumental activities of daily living (IADL).

## 6. Conclusions

There is evidence that the number of older people is rising, and that, among these, 50 million individuals in the world exhibit dementia, a number that is expected to increase to 152 million by 2050 [68]. On the one hand, the direct consequence of dementia is a reduction in the quality of life (e.g., because of the occurrence of depression and anxiety) of patients and their caregivers, and, on the other hand, this morbid condition represents a significant cost to national health systems that is predicted to be approximately USD 1 trillion per year [68]. This highlights the urgency of preventing dementia to the greatest degree possible. As reported elsewhere, physical inactivity is a significant risk factor for the occurrence of this age-related morbid condition, while older people with dementia report more physical problems than cognitively healthy controls [69]. Therefore, policymakers should prioritize the implementation of multifactorial interventions aimed at empowering physical health, cognitive functioning (e.g., through specific interventions aimed at boosting cognitive efficiency and strategies to improve learning and memory), and cognitive reserves (e.g., through more cognitively demanding leisure activities, such as reading, learning a second language, attending a course of creative writing) in midlife and late adulthood to prevent the occurrence of cognitive decline. Moreover, physical interventions should be promoted for individuals with dementia to preserve their residual cognitive functioning [69,70]. The current results suggest that implementation of systematic screening of the geriatric population to monitor developmental trends associated with the cognitive and motor functioning of older individuals, and for the early detection of those individuals needing multicomponent and personalized interventions, should be encouraged. The administration of a battery of cognitive and motor tasks, such as those used in the current investigation, together with self-report psychological well-being inventories (since depression is often associated with cognitive decline [69]), should be systematically used in clinical settings.

## Figures and Tables

**Table 1:** Zero-order correlations among global cognitive efficiency (i.e., MMSE), clock-drawing (i.e., clock), semantic fluency, ACE-R Verbal Fluency subtest (i.e., ACE-R Fluency), speed of processing and number sequencing (i.e., TMT-A), selective and divided attention (i.e., Attentional Matrices), Timed-Up-and-Go (i.e., TUG overall duration, TUG-Sit-to-stand, TUG-Stand-to-sit, TUG-Intermediate turn, TUG-Final turn), walking speed, step length and handgrip strength parameters.

	1	2	3	4	5	6	7	8	9	10	11	12	13	14
1 MMSE	—													
2 Clock	0.656 ***	—												
3 Semantic fluency	0.361 ***	0.393 ***	—											
4 ACE-R Fluency	0.658 ***	0.606 ***	0.458 ***	—										
5 Attentional Matrices	0.276 **	0.421 ***	0.295 **	0.441 ***	—									
6 TMT-A	−0.244 *	−0.443 ***	−0.170	−0.326 **	−0.552 ***	—								
7 TUG overall duration s	−0.128	−0.187	0.072	−0.164	−0.050	0.188	—							
8 TUG Sit-to-stand time s	−0.179 *	−0.231 *	−0.204	−0.180 *	−0.063	0.272 *	−0.012	—						
9 TUG Stand-to-sit time s	−0.189 *	−0.214 *	−0.222 *	−0.257 **	−0.196	0.182	0.418 ***	0.170	—					
10 TUG Intermediate-turn s	0.020	−0.088	0.221 *	−0.087	−0.037	0.048	0.702 ***	−0.330 ***	0.073	—				
11 TUG Final-turn s	0.051	−0.189	−0.001	−0.083	−0.054	0.235 *	0.554 ***	0.202 *	0.276 **	0.331 ***	—			
12 Walking speed m/s	0.081	−0.170	−0.133	0.020	−0.050	0.023	0.061	0.135	0.235 **	−0.178 *	0.125	—		
13 Step length m	−0.164	−0.060	−0.217 *	−0.083	−0.037	0.092	−0.131	0.154	0.053	−0.170	−0.193 *	0.211 *	—	
14 Handgrip strength (kg_f_)	0.226 *	0.215 *	−0.083	0.230 *	0.126	−0.321 **	−0.291 ***	−0.238 **	−0.264 **	−0.159	−0.232 **	−0.010	−0.034	—

Note. * *p* < 0.05, ** *p* < 0.01, *** *p* < 0.001.

**Table 2:** Summary of the logistic regression analysis for motor measures predicting the occurrence of cognitive impairment (0 = MCI, 1 = dementia), with background (BMI, gender, age, MMSE) variables controlled for.

						95% Confidence Interval	
Predictor	Estimate	SE	Z	*p*	Odds Ratio	Lower	Upper
Intercept	−9.26513	3.4358	−2.6967	0.007	9.47e-5	1.13e-7	0.0796
BMI	−0.14562	0.0768	−1.8958	0.058	0.864	0.744	1.0049
Gender	−0.03763	0.9271	−0.0406	0.968	0.963	0.156	5.9267
MMSE	0.56221	0.1056	5.3225	<0.001	1.755	1.426	2.1581
Handgrip strength	0.00485	0.0552	0.0880	0.930	1.005	0.902	1.1196
Walking speed	−0.11830	0.0580	−2.0409	0.041	0.888	0.793	0.9953
Step length	−0.20824	0.8112	−0.2567	0.797	0.812	0.166	3.9819
TUG Sit-to-stand	1.13580	0.5647	2.0112	0.044	3.114	1.029	9.4182
TUG Stand-to-sit	−0.29784	0.1321	−2.2546	0.024	0.742	0.573	0.9618

Note. Estimates represent the log odds of “Cognitive Impairment = MCI” vs. “Cognitive Impairment = Dementia”.

## Data Availability

The data that support the findings of this study are not publicly available due to privacy or ethical restrictions.
